# Use of Endobutton for Small Avulsion Fracture of Coronoid in the Terrible Triad of the Elbow: A Case Report

**DOI:** 10.7759/cureus.38119

**Published:** 2023-04-25

**Authors:** Ankur Salwan, Amit Saoji, Gajanan Pisulkar, Abhiram A Awasthi, Shounak Taywade

**Affiliations:** 1 Department of Orthopaedics and Traumatology, Jawaharlal Nehru Medical College, Datta Meghe Institute of Higher Education and Research, Wardha, IND; 2 Department of Orthopedics and Traumatology, Jawaharlal Nehru Medical College, Datta Meghe Institute of Higher Education and Research, Wardha, IND

**Keywords:** stiff elbow, endobutton, radial head fracture, coronoid fracture, terrible triad

## Abstract

The terrible triad (TT) of the elbow consists of coronoid process (CP) fracture, fracture of the radial head (RH), and posterior dislocation. Although the coronoid is an important anterior stabilizer, it is still unclear how to treat comminuted coronoid fractures. Poor fixation of the CP tends to result in posterolateral instability at the elbow joint and often in chronic instability. The ligamentous injuries also cause instability in elbow dislocations and should be suspected. There are various techniques available for coronoid fracture fixation. In this case report, we want to highlight our experience managing a 47-year-old male with posterior dislocation of the elbow after computed tomography (CT) confirmed that the patient had an RH fracture with an avulsion fracture of the coronoid. This TT of the elbow was managed with the help of an endobutton and a Herbert screw for coronoid avulsion fracture and RH fracture, respectively, through a lateral (Kocher) approach in our tertiary care hospital with satisfactory results. The use of endobutton is recommended in type 1 and type 2 coronoid fractures with no or minimal capsular attachment for good suspensory effect, and it emphasizes the possibility of associated coronoid fracture in case of posterior elbow dislocation. This case report emphasizes the fixation of even small fragments of the coronoid fracture for better stability and early mobilization. Postoperative rehabilitation involved using a hinged brace and early mobilization to avoid a stiff elbow and periodic X-rays to check the heterotopic ossification risk.

## Introduction

Terrible triad (TT) of the elbow consists of a combination of posterior dislocation of the elbow along with a fracture of the coronoid process (CP) of the ulna and radial head (RH) fractures [1]. The injury is caused by landing on an extended hand with a valgus force operating across it [2]. Although the CP of the ulna is an important anterior stabilizer, it is still unclear how to treat comminuted coronoid fractures. A TT of the elbow is labeled so because the injuries are severe and challenging to heal. It may lead to elbow stiffness, instability, and post-traumatic arthritis, which would impair function and cause persistent pain [3]. Poor fixation of the CP tends to result in posterolateral instability at the elbow joint and often in chronic instability. The ligamentous injuries also cause instability in elbow dislocations and should be suspected. There are various techniques available for coronoid fracture fixation. The four primary compartments of the elbow joint are as follows: (a) the anterior compartment, which is made up of the brachialis muscle, CP, and anterior capsule; (b) the lateral compartment, which is made up of the lateral condyle, lateral ligament, and capitulum; (c) the posterior compartment, which is made up of the olecranon, triceps muscles, and posterior capsule; and (d) the medial compartment, which includes the medial condyle, medial collateral ligament, and CP [4]. These structures all preserve joint stability. It is the most severe and difficult injury to treat due to the increased likelihood of instability and eventual arthrosis that is associated with an acute episode of instability [5]. This injury is brought on by high-velocity trauma, and it frequently leads to significant injury of the radial collateral ligament complex of the joint capsule and eventually reaches the ulnar collateral compartment. In this case report, we want to highlight our experience managing a 47-year-old male patient with TT of the elbow. This TT of the elbow was managed by close reduction and fixation with the help of an endobutton and a Herbert screw for coronoid avulsion fracture and RH fracture, respectively, through a lateral (Kocher) approach in our tertiary care hospital with satisfactory results. This case report emphasizes the fixation of even small fragments of the coronoid fracture for better stability and early mobilization in the case of TT elbow.

## Case presentation

**Figure 1:**
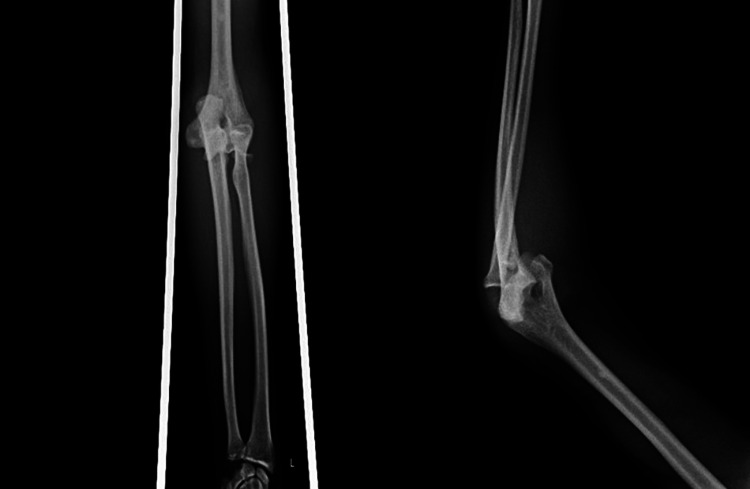
X-ray anteroposterior and a lateral view showing posterior dislocation of the elbow

A 47-year-old male patient presented to the emergency department with a history of falls from a height of 4 feet on his right upper limb eight days back. On examination, moderate swelling and ecchymosis were present over the medial side of the elbow. The olecranon process was prominent, and the three-point bony relationship was disturbed. The patient had a closed elbow fracture and dislocation. There was direct tenderness over the RH, and pronation-supination was painful. X-ray lateral and anteroposterior views of the elbow were done and were suggestive of posterior dislocation of the elbow with RH fracture (Figure 1).

**Figure 2:**
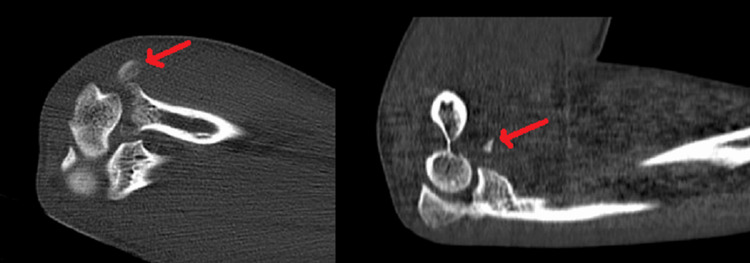
CT scan of the elbow joint showing fracture of the radial head and avulsion fracture of the coronoid (marked with red arrows) CT, computed tomography

**Figure 3:**
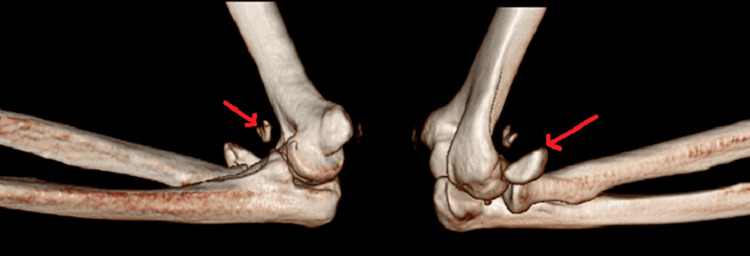
Three-dimensional reconstruction CT scan of the elbow joint showing avulsion fracture of the coronoid and fracture of the radial head (marked with red arrows) CT, computed tomography

The posterior dislocation was reduced in the emergency department, and on examination, the elbow was unstable beyond 50°-60° of extension. Examination revealed no neurovascular insufficiency, and an above-elbow slab was given to the patient. Further, computed tomography (CT) was done, which showed an avulsion fracture of the tip of CP that is a type 1 of the Regan-Morrey classification and RH fracture (Mason type 2 ) (Figures 2, 3).

Operative procedure

**Figure 4:**
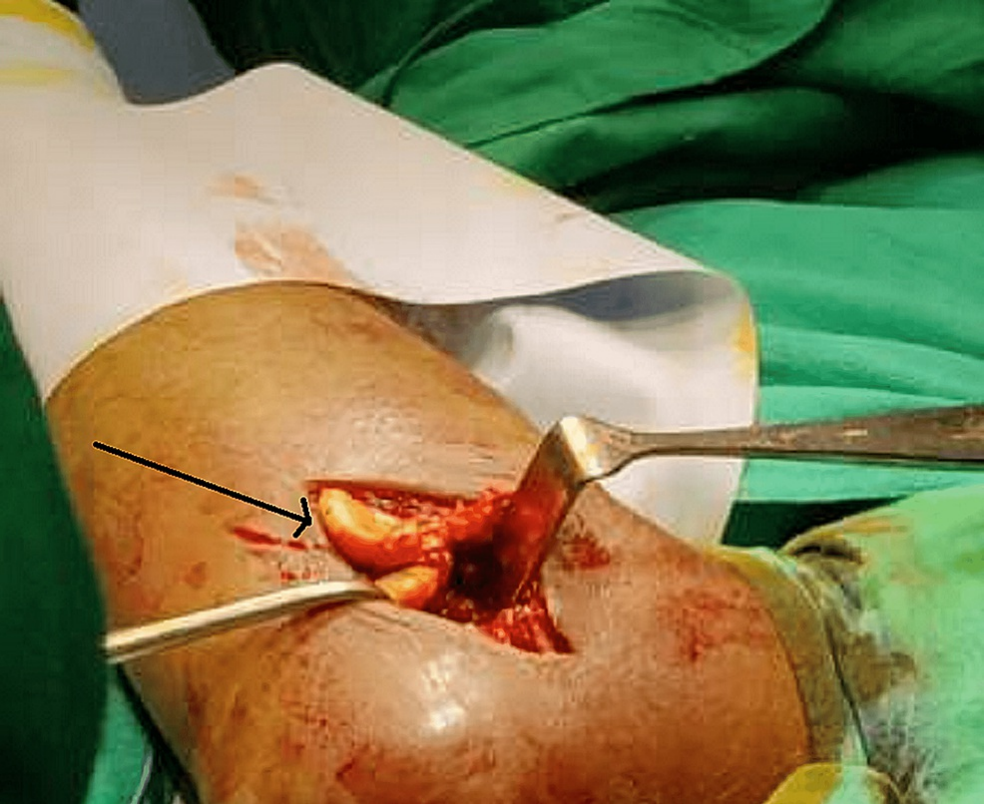
Fractured fragment of the radial head (marked with black arrow)

After the preoperative assessments, the patient was taken for the operation in a supine position over the operating theater (OT) table under peripheral axillary nerve block anesthesia. The tourniquet was placed over the proximal arm below the deltoid muscle after administration of a prophylactic antibiotic. A stockpile of surgical drape sheets was folded and placed behind the shoulder at the afflicted location while the patient was lying on the OT table in the supine position to fit the elbow in both flexed and extended positions. To address the RH fracture and coronoid fracture lateral (Kocher) approach, a lateral incision of 7 cm was taken. A lateral approach is used to utilize any preexisting tears in the common extensor origin, lateral capsule, or lateral ulnar collateral ligament. Pronation of the forearm is done to move the posterior interosseous nerve away from the incision. Kocher's interval was identified, and the RH exposure was done after cutting the extensor carpi radialis longus from its origin from the supracondylar ridge (Figure 4).

**Figure 5:**
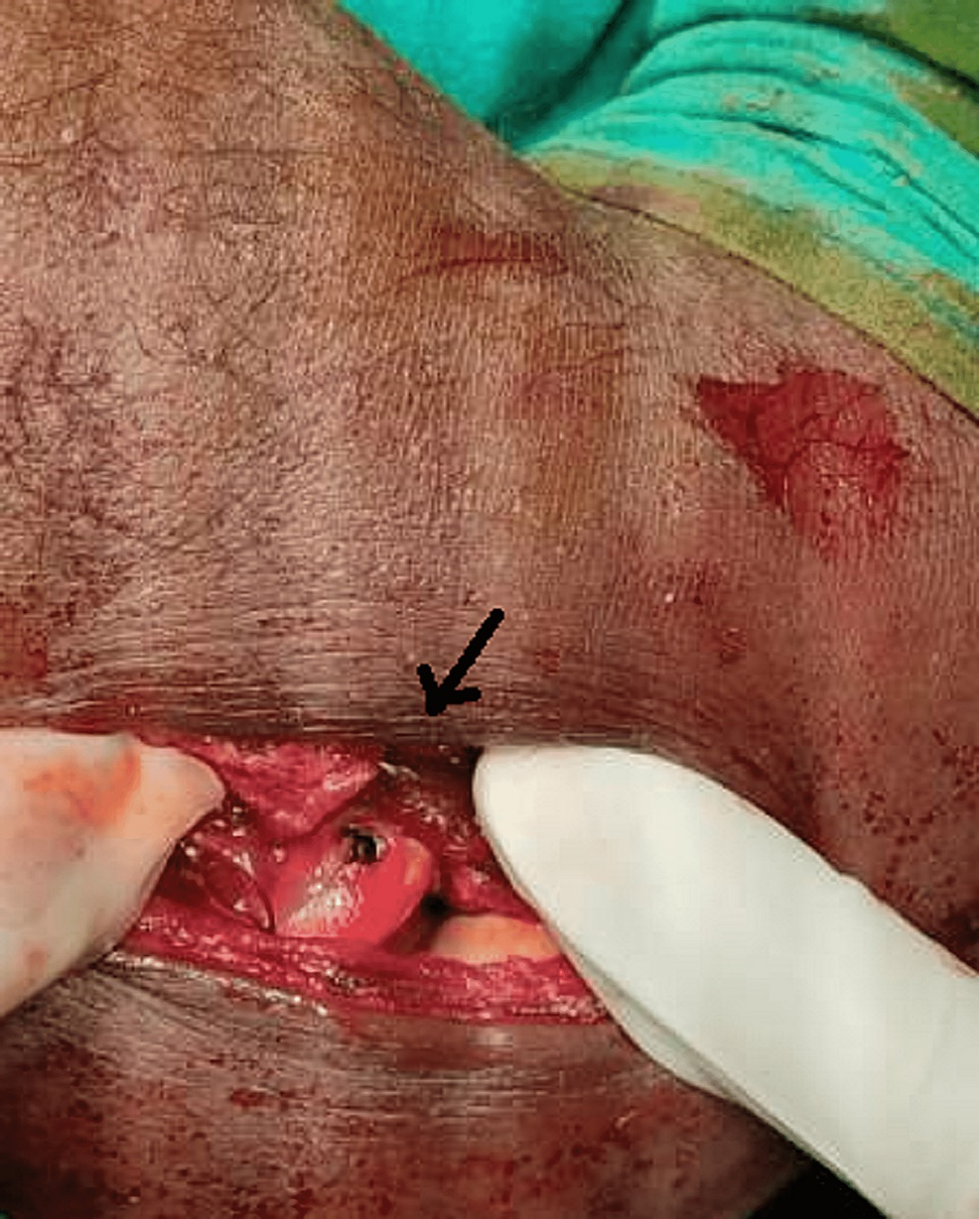
Radial head fracture fixed with Herbert screw (marked with black arrow)

The coronoid was accessed laterally after moving the RH fracture fragments. We chose to repair the CP fracture with an endobutton when we discovered that the anterior part of the capsule had been avulsed from its attachment. An endobutton was placed over the fracture and capsule after the capsule was meticulously repaired. Endobutton provides a suspensory effect and a great surface area to hold the fragments. Additionally, sutures are inserted via the loose capsular attachment to produce an anchor effect, and they are later extracted through the opening of the endobutton. Two parallel 2.0-mm K-wire holes were drilled through the posterior portion of the proximal ulna. Endobutton sutures were put into the holes with the use of suture passers. The sutures were tensioned to reduce the fragment and tied to the subcutaneous posterior border of the ulna. Coronoid fixation using the suture lasso approach will not result in a stable fixation if the anterior capsule's attachment to the coronoid pieces is disturbed or diminished with fragmentation. When fragmentation is visible and the pieces loosely adhere to the capsule, the suture lasso technique is unable to hold the little pieces together. Hence, there is a need to augment it with an endobutton. Also, there are chances of slippage of the knot in the suture lasso technique, so here the endobutton is slightly on the ulna border to hold the fragment in place. The elbow's lateral collateral ligament was repaired using a thick, braided, non-absorbable suture passing through bone tunnels, and the RH was fixed with a Herbert screw to secure it in place (Figure 5).

The fixation was confirmed, the closure was done in layers, and the patient was then sent to the postoperative department. Limb elevation with close monitoring of the swelling was done, and no negative suction drain was used.

Postoperative

**Figure 6:**
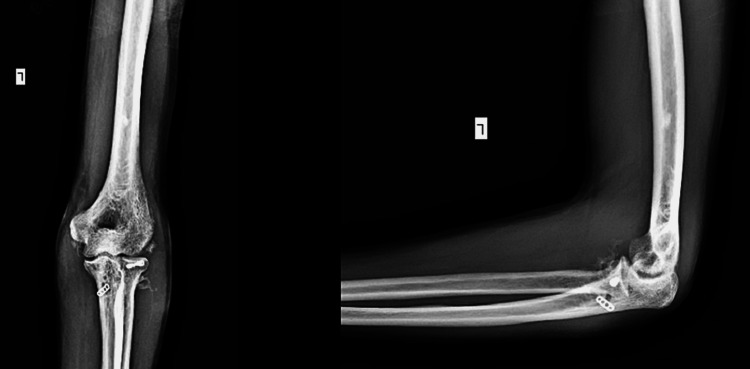
Eighteen months postoperative X-ray showing healed fracture of radial head and coronoid with no myositis

For the first 48 hours, the elbow was elevated and immobilized in flexion with an above-elbow slab in neutral rotation. An above-elbow hinged elbow brace was given to the patient on the third postoperative day. The flexor-pronator mass and common extensor origin, two muscle groups that function as dynamic stabilizers of the elbow, are recruited during active and active-assisted workouts for patients. The elbow must be in 90° of flexion to rotate the forearm fully. Exercises of the wrist and shoulders are recommended without restriction. For four weeks following surgery, we avoid the final 30° of extension, which is often the most unstable posture. For the first four weeks, indomethacin (100 mg daily) was used to treat discomfort and swelling as well as to prevent heterotopic ossifications (HO). The suture removal was done with a clean suture line. The hinged elbow brace was taken off six weeks after the procedure. The patient was allowed to resume some modest everyday activities for the first six weeks, but strengthening exercises and intensive activities were not allowed until this period. The X-ray from the follow-up examination at six months had been taken, and the X-ray after 18 months showed satisfactory union (Figure 6).

Up to six weeks, the maximum extension was limited to 30°. At 18 months, the patient had no evidence of myositis or HO and had average ranges of motion for the elbow and forearm rotation of 90° (10°-130°) and 110° (40°-160°), respectively.

## Discussion

The contribution of coronoid in the instability of the TT injury was first suggested by Josefsson et al. [6]. The integrity of the anterior capsule is crucial for elbow joint stability. Coronoid type 2 and type 3 fractures should be surgically corrected according to Regan and Morrey's classification and are linked to instability [7]. Since conservative treatment is ineffective and generally results in recurrent instability, surgery is the suggested course of action [8]. Because reduction contributes to the stability of the joint and even a small fragment of a minor coronoid fracture may be adhered to the anterior articular capsule, these fractures are clinically relevant [9]. Most CP fractures are associated with disruption of the anterior articular capsule [10]. Coronoid insufficiency may result in posterolateral rotatory instability. For TT, some authors also advocate the use of a posterior skin incision as both the medial and lateral aspects of the elbow can be accessed, and further skin incisions can be avoided. Despite being a rare complication in the context of trauma, flap necrosis is another potential problem [11]. Replacement of the RH or collateral ligament repair is less crucial than CP repair [12].

Pai and Pai, in their study, concluded that suture anchors are utilized for tiny and multiple fragmented coronoid fractures, while open reduction internal fixation with a plate and screw is used for big coronoid fragments [13]. Garrigues et al. demonstrate that the suture lasso fixing technique is better than the suture anchor for treating coronoid fractures, but it is useless for treating comminuted fractures [14]; hence, we used an endobutton to hold the fragments together. The suture lasso approach may not be sufficient to adequately cover all these fragments. As previously indicated, a material resembling a plate can keep these pieces together while simultaneously acting as a buttress. Additionally, with the assistance of the linked anterior capsule, we utilized the endobutton's suspensory function. A pre-contoured endobutton for sufficient covering can also improve this effect. As a result, the endobutton's buttress plate effect amplified the suspension effect. The anterior articular capsule can be partially repaired by reducing the tiny portion of the coronoid, which improves joint stability.

In the technique described, it is possible to place an endobutton in front of the coronoid pieces and suspend them via the tunnels. Because of this, the fragments could still be retained together even though they only have a slight attachment to the anterior capsule. The method we have outlined is recommended for individuals with type 1 and type 2 multi-fragmented coronoid fractures who present with instability. Additionally, it may be necessary to treat type 2 coronoid fractures since they contain tiny fragments of the anteromedial coronoid facet. Additionally, the anterior capsule attachment to these fragments may have been lost. This technique may be useful for stabilizing coronoid type 1 fracture because according to the study by Doornberg et al., the majority of CP fractures in severe triad elbow injuries are type 1 fractures [12]. Hence, they require fixation to provide a stable joint.

## Conclusions

The management of TT elbow, a highly complex pattern of elbow instability, continues to be challenging. The true nature of RH and CP fractures may not be obvious on normal radiographs, so CT scanning is an invaluable supplement to diagnosis. The goal of early mobilization remains the same: to establish a stable elbow joint and to regain the functional arc in pain-free motion. Type 1 coronoid fractures fixed in this study result in early mobilization. The elbow joint was stable with a satisfactory functional arc. Repairing the anterior capsule and the damaged CP tip results in a more stable elbow, and fixing the fractured RH and employing the suspensory action of an endobutton in type 1 and type 2 CP fractures produce positive results and a prompt return to activity. Comminuted fractures of the coronoid that are difficult to stabilize using a suture loop or suture anchors can be treated with endobutton fixation.

## References

[1] Hildebrand KA, Patterson SD, King GJ (1999). Acute elbow dislocations: simple and complex. Orthop Clin North Am.

[2] Hotchkiss RN (1996). Fractures and dislocations of the elbow. Rockwood and Green's Fractures in Adults. 4th ed.

[3] Josefsson PO, Gentz CF, Johnell O (1987). Surgical versus non-surgical treatment of ligamentous injuries following dislocation of the elbow joint. A prospective randomized study. J Bone Joint Surg Am.

[4] Desai MM, Sonone SV, Badve SA (2006). The terrible triad of the elbow: a case report of a new variant. J Postgrad Med.

[5] O’Driscoll SW, Jupiter JB, King GJW, Hotchkiss RN, Morrey BF (2000). The unstable elbow. J Bone Jt Surg Am.

[6] Josefsson PO, Gentz CF, Johnell O, Wendeberg B (1989). Dislocations of the elbow and intraarticular fractures. Clin Orthop Relat Res.

[7] Regan W, Morrey B (1989). Fractures of the coronoid process of the ulna. J Bone Joint Surg Am.

[8] Ring D, Jupiter JB, Zilberfarb J (2002). Posterior dislocation of the elbow with fractures of the radial head and coronoid. J Bone Joint Surg Am.

[9] Cage DJ, Abrams RA, Callahan JJ, Botte MJ (1995). Soft tissue attachments of the ulnar coronoid process. An anatomic study with radiographic correlation. Clin Orthop Relat Res.

[10] Terada N, Yamada H, Seki T, Urabe T, Takayama S (2000). The importance of reducing small fractures of the coronoid process in the treatment of unstable elbow dislocation. J Shoulder Elbow Surg.

[11] Mathew PK, Athwal GS, King GJ (2009). Terrible triad injury of the elbow: current concepts. J Am Acad Orthop Surg.

[12] Doornberg JN, van Duijn J, Ring D (2006). Coronoid fracture height in terrible-triad injuries. J Hand Surg Am.

[13] Pai V, Pai V (2009). Use of suture anchors for coronoid fractures in the terrible triad of the elbow. J Orthop Surg (Hong Kong).

[14] Garrigues GE, Wray WH 3rd, Lindenhovius AL, Ring DC, Ruch DS (2011). Fixation of the coronoid process in elbow fracture-dislocations. J Bone Joint Surg Am.

